# Calcium Supplementation- Efficacy and Safety

**DOI:** 10.1007/s11914-025-00904-7

**Published:** 2025-02-12

**Authors:** Ian R. Reid

**Affiliations:** Department of Medicine, Faculty of Medical and Health Sciences, University of Auckland, Private Bag 92019, Auckland, New Zealand

**Keywords:** Osteoporosis, Fracture, Bone mineral density, Myocardial infarction, Cardiovascular disease, Renal calculi, Mendelian randomization

## Abstract

**Purpose of Review:**

To assess the efficacy of calcium supplements in preventing fractures, and to review their adverse effects, particularly on the cardiovascular system.

**Recent Findings:**

There is now a large body of trial evidence demonstrating that calcium supplements do not prevent fractures in community-dwelling adults. They commonly produce gastrointestinal side-effects, sometimes serious, and increase the risk of renal calculi. Meta-analyses of adverse events from clinical trials suggest that the risk of MI is increased by 10–20% with calcium supplementation, though dietary calcium intake does not appear to be a cardiac risk factor. Ingestion of a calcium bolus increases circulating calcium concentrations for the following 8 h, accompanied by acute increases in blood coagulability and calcification propensity, with blood pressures > 5 mmHg higher than placebo-treated individuals. Mendelian randomization studies demonstrate that circulating calcium levels are a significant risk factor for cardiovascular disease, so the acute calcium-elevating effect of supplements might contribute to increased cardiovascular risk.

**Summary:**

The current balance of evidence suggests that calcium supplements have little role in the prevention or treatment of osteoporosis, since estrogen and bisphosphonates prevent fractures without their co-administration. Specific studies are needed to address whether calcium is benficial with anabolic bone medicines.

## Introduction

The practice of managing osteoporosis with calcium supplements developed at a time when evidence-based medicine in this area was practically non-existent and when detailed knowledge of bone micro-anatomy and cell biology were only rudimentary. Bone density measurements at common fracture sites were not possible until the late 1980s, so calcium balance was used as a surrogate for the assessment of the effects of calcium on skeletal strength. When bone densitometry became available and could be used to assess the effects of calcium supplementation, small positive effects were found [1], but much smaller than we now recognize are needed for effective fracture prevention [2]. Shortly after the first axial bone mineral density (BMD) studies in the early 90s, Chapuy et al. [3] published their landmark paper demonstrating that calcium plus vitamin D (Ca + D) reduced fracture rates in elderly women living in rest homes. This study in women who were severely vitamin D deficient was generalized to the management of osteoporosis in non-D-deficient populations, and so became the standard of care used in the randomized controlled trials of medications for fracture prevention that followed in the mid-1990s and subsequently. This has led to the statement that anti-resorptive drugs do not work unless given with calcium and vitamin D, an assertion that now has been tested repeatedly and found to be incorrect (discussed below).

With the new millennium, came multiple randomized controlled trials of the effects of calcium supplements on BMD and fracture, which showed the BMD effects were real but small and non-cumulative, and that fracture effects in community-based studies were not detectable. At the same time, the adverse event data from these trials indicated that calcium supplements were not without risk. More recently, these risks have been studied further, particularly those of cardiovascular disease. In this regard, the physiological effects of calcium supplements have been explored, particularly on circulating calcium concentrations, and the impact of small changes in these concentrations on vascular health have been described. This review will summarize recent progress across these areas.

## Bone Structure and Biology

Bone is a connective tissue, its structure defined by bundles of type I collagen fibrils each about 50 nm in diameter [4]. Between the collagen fibrils are long thin (about 5 nm) plates of hydroxyapatite known as mineral lamellae, which give bone its rigidity. Type 1 collagen in bone is laid down by bone-forming cells (osteoblasts) and remodeled by bone-resorbing cells (osteoclasts). The activities of osteoblasts and osteoclasts are regulated by hormones and cytokines, and the balance of these cellular activities determines changes in bone mass over time. Normal extracellular fluid concentrations of calcium and phosphate are necessary for hydroxyapatite plates to form, producing a mineralized tissue. While mineralization is critical to normal bone function, mineral deposition in other tissues (e.g. cardiovascular, renal) can be very deleterious. To prevent this, the body produces pyrophosphate, a mineralization inhibitor. Osteoblasts produce alkaline phosphatase which breaks down pyrophosphate, permitting local skeletal mineralization. Thus, a delicate balance must be maintained such that mineralization can take place in bone but not elsewhere. The ingestion of large boluses of calcium or phosphate has the potential to upset this balance.

## How Much Calcium Do We Need?

In the past, this question has been assessed using calcium balance studies, a technique which assesses intakes and losses (urinary and fecal) of calcium, and assumes that the balance of these fluxes represents a net gain or loss of bone. Studies in the 1930s to 1950s demonstrated the achievement of calcium balance with intakes between one hundred and several hundred mg/day [5–9]. Intakes at this level have been common in Africa and Asia and are not associated with bone pathology in adults, though intakes below about 200 mg/day do result in rickets in some children [10]. Accordingly, in 1974 the Food and Agriculture Organization and the World Health Organization recommended minimum intakes of calcium for adults of 400–500 mg/day [11]. Later that decade, Heaney et al. published two analyses of calcium balance studies in 168 perimenopausal nuns, demonstrating that calcium balance was directly related to calcium intake and that zero balance could be achieved in premenopausal women at an intake of about 990 mg/day and in postmenopausal women with 1500 mg/day [12, 13]. These findings have been very influential in determining recommended calcium intakes in America, but were based on a flawed analysis. Because balance is the difference between intake and output, there is a mathematical inevitability that a positive relationship will be found between balance and intake even if there is no biological basis for it. Subsequent observational studies and clinical trials of calcium supplementation have not demonstrated that postmenopausal bone loss can be halted with these calcium intakes [14–16]. More recent calcium balance studies in adults which avoided the statistical error of the Heaney studies concluded that ”calcium balance was highly resistant to a change in calcium intake across a broad range of typical dietary calcium intakes (415–1,740 mg/day…)” [17]. A 2016 analysis of balance studies in adults aged < 60 years from China demonstrated positive calcium balances with intakes as low as 300 mg/day [18]. Despite these recent data, many guidelines remain heavily influenced by the Heaney studies, and recommend higher intakes than are justified by the more recent data.

The advent of whole body DXA scanning has rendered calcium balance studies obsolete for assessment of the impact of calcium intake on bone health. It is now possible to measure bone balance (i.e. change in total body bone mineral) over periods of years, rather than just the days to weeks assessed in calcium balance studies. We have conducted two studies of the relationships between calcium intake and change in total body bone mineral, each in > 1000 postmenopausal women, over periods of 5 and 6 years. Calcium intakes ranged from 300 mg/day to 2000 mg/day. Both studies demonstrated ongoing loss of total body bone mineral which was unrelated to calcium intakes across the study periods [14, 15]. A similar 2-year study in older men also showed no effect of calcium intake on bone balance [19]. These findings suggest that calcium intakes of > 500 mg/day are likely to be adequate in adults, consistent with the United Nations recommendations from the 1970s. These findings are congruent with the absence of a relationship between calcium intake and BMD in adults aged > 50 years in the NHANES study [20], and with the absence of an association between calcium intake and fracture risk in meta-analyses of observational studies [21, 22]. It is notable that neither the FRAX nor Garvan calculators use calcium intake as a predictor of fracture risk.

Clearly, calcium balance studies and those based on bone density measurements give different answers regarding dietary calcium requirement. BMD and changes in BMD predict fractures, which has not been demonstrated for calcium balance. Balance studies are too short-term to permit homeostatic adjustment to changes in dietary calcium intake [23, 24] and they assume that there is no net uptake of calcium into soft tissue, which is not the case in older adults with cardiovascular or renal disease [25]. For these reasons, calcium requirements should not be estimated based on balance studies, and the value of calcium supplements should be determined from randomized trials of their effects on BMD and fractures.

**Fig. 1:**
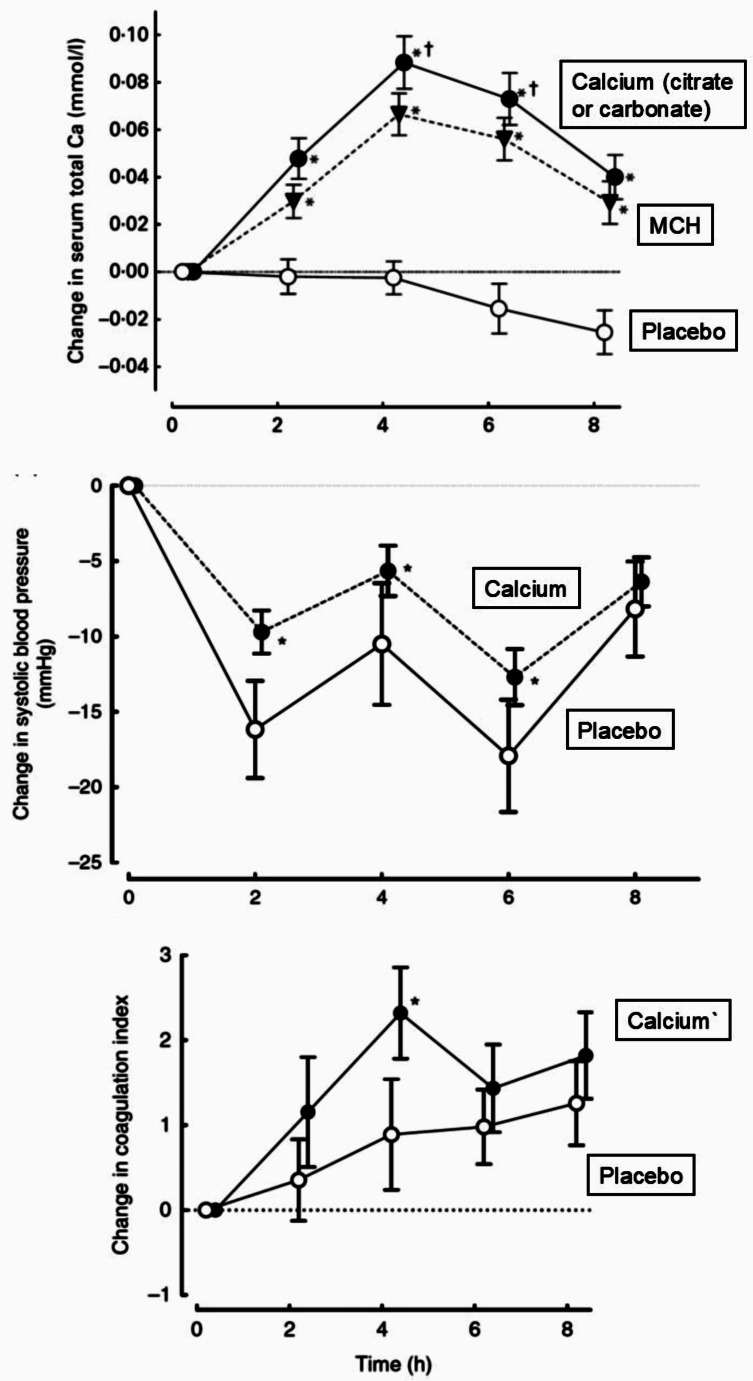
Effects of the ingestion of 1 g calcium as citrate, carbonate or microcrystalline hydroxyapatite (MCH), compared with placebo, on serum calcium, systolic blood pressure, and blood coagulation measured by thromboelastography in healthy postmenopausal women (*N* = 96). The calcium and MCH groups were pooled for the blood pressure data and the coagulation index was only assessed in the calcium citrate and placebo groups. Data are means and standard errors. Changes in serum calcium were significantly different between the 3 groups shown (*P* < 0.0001), changes in systolic blood pressure were significantly different between the calcium and placebo groups between 2 and 6 h (all *P* < 0·02), and coagulation index was elevated above placebo at 4 h (*P* = 0.03). The blood pressure effects were reproduced in a subsequent study [31]. This study demonstrates that a calcium supplement has effects on these parameters that could adversely influence cardiovascular risk. From Bristow et al. [27, 30],, Copyright 2015, Cambridge University Press, re-used with permission

## Acute Effects of Calcium Supplements

When calcium is ingested as part of a mixed meal it has very little impact on bone metabolism since eating is itself associated with an acute reduction in bone resorption [26], and the calcium component of the meal (likely to be < 100 mg) is absorbed slowly as a result of slow gastric emptying after ingestion of fat and protein. In contrast, a 500–1000 mg calcium supplement, particularly if taken fasting, results in the rapid absorption of calcium with elevation of circulating calcium levels to the upper end of the normal range, a change that persists for at least 8 h (Fig. 1) [27]. Secretion of parathyroid hormone is suppressed acutely, and remains lower long-term, resulting in a ~ 20% reduction in bone turnover markers [28]. These acute changes in plasma calcium concentrations are associated with acute increases in blood coagulability and calcification propensity, as well as blood pressure levels > 5 mmHg higher than placebo-treated individuals over the 6 h after calcium ingestion (Fig. 1) [29–31]. This contrasts with findings 12–24 h after dosing, when users of calcium supplements have reductions in systolic blood pressure of 1–2 mmHg, which do not persist with long-term calcium use [32]. Thus, calcium supplements produce easily detectable physiological changes that extend beyond the skeleton. Those affecting blood pressure and the physical properties of blood might be relevant to the possible adverse effects of calcium supplements on cardiovascular events, a discussed below.

## RCTs of Calcium Supplementation

### BMD

Consistent with the acute effects of supplements on circulating parathyroid hormone levels and on markers of bone turnover, supplements do have small positive effects on BMD. Trials demonstrate between-groups differences of about 1% in total hip BMD, and this effect is the same whether trial duration is 1 year, 2 years, or ≥ 3 years [33]. This indicates that the weak antiresorptive effect of a calcium supplement leads to a filling in of bone remodelling space but not to a sustained change in rates of bone loss. Recent meta-regression analyses of individual patient data from almost all trials of fracture prevention have demonstrated that a between-groups difference in change in total hip BMD of at least 2.1% at 2 years is required for prevention of non-vertebral fracture and of 3.2% for prevention of hip fracture [2, 34]. Thus, the BMD effects of calcium supplements would not be expected to result in anti-fracture efficacy. Trials in premenopausal women have not found clinically significant effects on BMD [35].

**Table 1:** Major trials of calcium ± vitamin D on fracture

Study	Setting	*N*	Age (years)	25OHD (nmol/L)	Calcium (mg/d)	Vitamin D (IU/d)	Duration (months)	Relative Risk of Fracture	
								Total	Hip
Chapuy 1992,1994	Nursing home	3270	84 (6)	20 (14)	1200	800	36	**0.83 (0.71, 0.97)**	**0.77 (0.62, 0.96)**
RECORD 2005	Community	5292	77 (6)	38 (16)	1000	800	45	0.93 (0.82, 1.06)	1.10 (0.83, 1.47)
Porthouse 2005	Community	3314	77 (5)	-	1000	800	25	0.96 (0.70, 1.33)	0.71 (0.31, 1.64)
Women’s Health Initiative 2006	Community	36,282	62 (7)	48 (24)	1000	400	84	0.97 (0.92, 1.03)	0.88 (0.72, 1.07)
Prince 2006	Community	1460	75 (3)	77	1200	0	60	0.87 (0.69, 1.10)	1.83 (0.68, 4.93)
Reid 2006	Community	1471	74 (4)	52 (19)	1000	0	60	0.92 (0.75, 1.14)	**3.43 (1.27, 9.26)**
Salovaara 2010	Community	3432	67 (2)	50 (18)	1000	800	36	0.83 (0.62, 1.11)	2.00 (0.37, 10.88)

Baseline age and 25-hydroxyvitamin D (25OHD) data are mean (SD). Relative risks are given with 95% confidence intervals. Where the confidence intervals do not include 1, data are bolded. Study participants were all female, except in the RECORD study, which was 15% male. Data from Bolland et al., 2015 [22]. Copyright IR Reid, used with permission

**Table 2:** Association of genetically predicted serum calcium with cardiovascular disease in mendelian randomization studies

Reference	Source	Cases	Controls	OR (95%CI)	*P*	Unit of Effect
**Coronary Artery Disease**						
Larsson, 2017 [64]	CardiogramplusC4D	60,801	123,504	1.25 (1.08, 1.45)	0.003	1 SD (0.5 mg/dL)
Xu, 2017 [82]	CardiogramplusC4D	60,801	123,504	1.49 (1.02, 2.17)	< 0.05	1 mg/dL
Yuan, 2022 [65]	UK Biobank	24,571	288,306	1.21 (1.09, 1.33)	0.0003	1 SD
**Myocardial Infarction**						
Zhou, 2019 [83]	UK Biobank	9,828	311,419	1.99 (1.17, 3.39)	0.011	1 mg/dL
Xu, 2017 [82]	CardiogramplusC4D	43,676	128,199	1.58 (1.06, 2.35)	< 0.05	1 mg/dL
Zhou, 2019 [83]	CardiogramplusC4D	43,676	128,199	1.48 (1.08, 2.02)	0.015	1 mg/dL
Yuan, 2022 [65]	UK Biobank	13,108	288,306	1.31 (1.14, 1.50)	0.0001	1 SD

OR = odds ratio, and are shown either per standard deviation (SD) or per mg/dL of serum calcium. Where different investigators have both analyzed the same database, they have used a different number of single-nucleotide polymorphisms to predict serum calcium so produced slightly different ORs

Analysis of data from the FinnGen Consortium with 30,952 cases of coronary artery disease and 11,622 cases of myocardial infarction produced ORs very similar to those from CardiogramplusC4D but are only reportedly graphically [65], so exact figures are not available to include in this table. Copyright IR Reid, used with permission

### Fracture

As noted in the Introduction, one trial of Ca + D in vitamin D-deficient nursing home residents demonstrated fracture prevention [3, 36], but those women were severely vitamin D deficient with mean 25-hydroxyvitamin D levels in the placebo group during the study of 14 nmol/L, when adjusted to contemporary assay standards [37]. However, fracture prevention is not found in the much larger number of studies carried out in the community. The most comprehensive meta-analysis of community-based studies concluded that “the use of supplements that included calcium, vitamin D, or both compared with placebo or no treatment was not associated with a lower risk of fractures among community-dwelling older adults” [38]. This analysis included 11 studies of calcium alone, 16 of vitamin D alone, and 10 with the combined supplements as the intervention. This view has been adopted by the United Sates Preventive Services Task Force [39, 40] and the International Osteoporosis Foundation [41]. The major studies in this area are summarised in Table 1, and make clear the uniqueness of the Chapuy study in terms of both the characteristics of its participants and its outcomes. Some previous meta-analyses not limited to community-dwelling individuals have included this study and their results have been dominated by the size of the Chapuy trial. Generalizing findings from this special population to the broader community is not appropriate. The finding that calcium supplement use causes small effects on loss of BMD but not on fracture rates was also reported recently from the SWAN study [42].

Contrasting with the community studies, the Chapuy study demonstrates the importance of preventing and treating vitamin D deficiency in the frail elderly. Further, the recent study of Iuliano et al. indicates that deficiency of other nutrients might also contribute significantly to skeletal fragility in the institutionalized elderly [43]. In a cluster randomised controlled trial, 60 residential care homes were randomized to provide residents with additional dairy products containing 560 mg calcium and 12 g protein daily. Residents already had a calcium intake of 600–700 mg/day and were vitamin D replete. Over 2 years, the intervention significantly reduced the risk of all fractures by 33%, hip fractures by 46%, and falls by 11%. The failure of trials of calcium alone to produce such an effect indicates that this finding was a result of a broad improvement in nutrition rather than attributable to any single nutrient.

Despite the broad agreement that supplements of calcium or vitamin D in the community do not reduce fracture risk, many doctors still provide these supplements to patients receiving pharmaceutical management of osteoporosis, on the basis that they were provided in the pivotal trials of most anti-osteoporosis medications. This is probably unnecessary for most agents, and calcium is likely to be a significant contributor to adverse events. The effects of alendronate on BMD are not increased by co-administration of calcium [44]. Trials of estrogen [45, 46] and the bisphosphonates, clodronate and zoledronate [47, 48], have demonstrated anti-fracture efficacy without calcium supplementation comparable to that achieved with its use. Certainly, hypocalcemia can occur if potent antiresorptive drugs are given to people who are severely vitamin D deficient, but vitamin D supplementation is what is needed to prevent this problem. The use of anabolic anti-osteoporosis medications without calcium supplementation has not been systematically assessed, so specific studies are needed to address this question.

## Calcium Supplements and Cardiovascular Disease

The propensity for diseased arteries to undergo calcification has long been recognized, but the impact of calcium supplements on cardiovascular health remains controversial. However, the general acceptance that these supplements do not make a significant contribution to fracture prevention has made this issue somewhat less important since, if there is no demonstrable benefit, then any risk is unacceptable. Many observational studies have related dietary calcium intake and/or supplement use to cardiovascular risk [49–54] or to progression of coronary artery calcification [55], with mixed results. While some studies suggest an adverse effect of calcium supplements, there is little evidence that dietary calcium has an adverse effect [50, 53, 56]. These studies are difficult to interpret because of potential confounding. In light of the prolonged increase in circulating calcium that follows supplement ingestion, there has been interest in whether plasma calcium levels impact on cardiovascular health.

## Observational Studies of Circulating Calcium and Cardiovascular Risk

A review of the association of circulating calcium concentrations and cardiovascular disease found 13 relevant studies, 11 being prospective cohort studies [57]. Eight studies reported a positive, statistically significant relationship between serum calcium and myocardial infarction (MI) or coronary heart disease. Meta-analysis found an 8% increase in risk of cardiovascular events per standard deviation increase in serum calcium concentrations, with a similar impact on death (hazard ratio [HR] 1.13/SD increase in serum calcium) [57]. Higher circulating calcium levels within the normal range are associated with increased intima-media thickness in the carotid arteries [58, 59], a higher prevalence of abdominal aortic calcification [60], and more calcified plaque in the coronary arteries [61, 62]. These findings, together with the data reviewed above demonstrating increases in serum calcium of about 1 SD for at least 8 h after ingestion of calcium supplements, make it possible that boluses of calcium might have an adverse effect on cardiovascular risk. This is further supported by their acute adverse effects on blood pressure and the coagulability and calcification propensity of blood, described above (Fig. 1) [29–31]. Interestingly, the closely related ion, strontium, has a similar adverse effect on myocardial infarction [63].

## Mendelian Randomization

The adverse associations of higher circulating calcium concentrations with vascular risk in observational studies might be confounded by various biases, but these findings have now been reinforced by multiple Mendelian randomization studies. This technique has been used to test for an association between genetic variants linked to higher circulating calcium levels and cardiovascular risk. With this technique, it can be inferred that any associations found are likely to be causal. Using Mendelian randomization, Larsson found that an increase in serum calcium of 1 standard deviation ( about 0.1 mmol/L) was associated with a 25% increase in the risk of MI [64]. Recent meta-analyses of Mendelian randomization studies have found that a higher serum calcium concentration is causally associated with increased risk of coronary artery disease (especially MI) and renal calculi, while not impacting on BMD or fracture risk [65, 66] (Table 2). Genetically predicted calcium level has also been shown to be associated with blood pressure, consistent with the acute effects of calcium administration described above [67].

## RCTs and Meta-Analyses

More direct evidence of an adverse cardiovascular effect of calcium supplements came from the Auckland Calcium Study, where there was a significant increase in the risk of MI in those randomized to calcium [68]. This finding led to our reviewing all other trials of calcium supplementation with available cardiovascular event data [69]. Upward trends in incidence of MI were present in most studies, with the meta-analysis showing a 27% increase in risk of MI. These findings were broadly confirmed by others. For calcium monotherapy, Lewis found a risk ratio for MI of 1.37 (0.98, 1.92) [70], Mao reported an odds ratio for MI of 1.28 (0.97–1.68) [71], and Yang a relative risk for coronary heart disease of 1.20 (1.08, 1.33) [56].

This matter has most recently been re-examined by Huo et al. [72]. They carried out a meta-analysis of the effects of calcium supplementation alone, following the pattern of our own previous study. However, they excluded trials with fewer than 500 people and they also excluded the RECORD study comparison between calcium plus vitamin D with vitamin D alone. These exclusions reduced the power of their analysis. They used a revised version of the Prince data, which appears to only include MIs that were recorded as the principal diagnosis during a first-time hospitalisation for an atherosclerotic event [73], rather than all documented MIs, removing the adverse trend in data they provided to us from that study [69]. The Huo analysis reported a relative risk of MI of 1.15 (0.88, 1.51) and of coronary heart disease death of 1.24 (0.89, 1.73) [72]. In patients with chronic kidney disease, calcium-based phosphate binders are associated with an increased risk of all-cause mortality when compared with non-calcium-based binders in clinical trials [74].

Conclusions are much less consistent when studies of calcium plus vitamin D (Ca + D) supplementation are assessed. This is principally because of the dominance of the Women’s Health Initiative (WHI) in these analyses as a result of its size (*N* = 36000). Analysis of the whole dataset does not show an increase in risk of MI with Ca + D, though there is a significant interaction between obesity and risk of MI - the HR for MI in those randomized to Ca + D is 1.17 in non-obese participants [75]. The WHI was unusual in that more than half of participants were taking calcium supplements at recruitment and continued to do so throughout the study. This has the potential to obscure effects of the treatment. Therefore, we carried out an analysis of the 16,000 participants who were not using calcium at the time of randomization, and found a hazard ratio for clinical MI of 1.22 (1.00, 1.50) in those randomized to Ca + D [76], similar to what we had reported in trials of calcium alone. Using this version of the WHI data, our meta-analysis of trials of calcium with or without vitamin D found a relative risk of MI of 1.24 (1.07, 1.45) and of stroke of 1.15 (1.00, 1.32) [76]. Thus, the apparent effects of Ca + D on MI depend on how the WHI data are handled. With extended follow-up of the WHI trial cohort to 22 years, cardiovascular mortality was significantly increased in those randomized to Ca + D (HR 1.06 [1.01, 1.12]) though this was counterbalanced by a decrease in cancer mortality (HR 0.93 [0.87, 0.99]) [77].

## Other Safety Considerations with Calcium Supplementation

Gastrointestinal side-effects, mainly bloating and constipation, have long been recognized as common consequences of calcium supplementation and they contribute to poor adherence with these agents [1]. A meta-analysis has shown that these complaints are increased 43% with calcium compared with placebo [78]. A recent clinical trial also reported that hospital admissions for acute abdominal problems were doubled by randomization to calcium [78]. Thus, the gastrointestinal side-effects of calcium are common, and can sometimes result in significant morbidity. Calcium supplements are bulky and difficult to swallow, sometimes causing choking [79].

Renal calculi are a second widely accepted effect of calcium supplements, consistent with the Mendelian randomization finding that higher circulating calcium levels are linked with this problem [65, 66]. In a study that supplemented calcium intake to 1200 mg/day in postmenopausal women, 9% became hypercalcemic and 31% hypercalciuric [80]. This is consistent with the WHI finding that randomization to Ca + D was associated with a 17% increase in renal calculi [81]. Calcium supplements are contra-indicated in those with a history of renal calculi.

## Conclusions

Calcium supplements produce small, non-cumulative effects on bone density which are not large enough to result in fracture prevention in clinical trials. Accordingly, their use in community-dwelling adults for fracture prevention is not supported by the US Preventive Services Task Force. They commonly produce gastrointestinal side-effects, sometimes serious, and increase the risk of renal calculi. Meta-analyses of adverse events from clinical trials suggest that the risk of MI is increased by 10–20% with calcium supplementation, though dietary calcium does not appear to be a cardiac risk factor. Mendelian randomization studies show that higher genetically predicted circulating calcium levels are associated with an increased risk of cardiovascular disease. Accordingly, most guidelines recommend that calcium should come from the diet, in preference to supplements. Despite this evidence base, some doctors continue to provide calcium as an adjunct to anti-osteoporosis medicines, though the anti-fracture efficacy of estrogen and bisphosphonates has been demonstrated to be maintained without calcium. Specific studies are needed to address whether calcium is needed with anabolic bone medicines. The current balance of evidence suggests that calcium supplements have little role in the prevention or treatment of osteoporosis.

## Key References

- Zhao J, Zeng X, Wang J, Liu L. Association between calcium or vitamin D supplementation and fracture incidence in community-dwelling older adults: A systematic review and meta-analysis. JAMA. 2017;318(24):2466-82.
  - Meta-analysis demonstrating that calcium supplements do not reduce fracture risk in community-dwelling adults.
- Bristow SM, Horne AM, Gamble GD, Mihov B, Stewart A, Reid IR. Dietary Calcium Intake and Bone Loss Over 6 Years in Osteopenic Postmenopausal Women. J Clin Endocrinol Metab. 2019;104(8):3576-84.
  - An important study demonstrating the independence of bone balance from calcium intake over 6 years in a large group of healthy postmenopausal women, reinforcing the findings of the meta-analysis of RCTs cited above.
- Larsson SC, Burgess S, Michaëlsson K. Association of Genetic Variants Related to Serum Calcium Levels With Coronary Artery Disease and Myocardial Infarction. JAMA. 2017;318(4):371 − 80.
  - A critical study using the novel technique of Mendelian randomization, to demonstrate that higher serum calcium levels within the normal range are causally associated with increased cardiovascular risk. This observation has now been confirmed in several subsequent analyses using this technique.
- Bristow SM, Gamble GD, Stewart A, Horne AM, Reid IR. Acute effects of calcium supplements on blood pressure and blood coagulation: secondary analysis of a randomised controlled trial in post-menopausal women. Brit J Nutr. 2015; 114:1868–1874.
  - Demonstration of significant acute adverse effects of calcium supplements on blood pressure and blood coagulation, providing a possible mechanism of the adverse effect of higher calcium level suggested by the Mendelian randomization studies.

## Data Availability

No datasets were generated or analysed during the current study.
